# Investigating risk factors and developing a predictive model for venous thromboembolism in patients with sepsis

**DOI:** 10.3389/fmed.2026.1890418

**Published:** 2026-10-05

**Authors:** Yu Han, Yage Shi, Liu Liu, Bingjie Ding, Mengjuan Li, Ao Xia, Jingyuan Liu, Juanjuan Song, Ao Xie, Dong Miao, Hu Zhou

**Affiliations:** 1 Department of Hematology, The Affiliated Cancer Hospital of Zhengzhou University and Henan Cancer Hospital, Zhengzhou, China; 2 Department of Hematology, Huaihe Hospital of Henan University, Kaifeng, China; 3 Department of Hematology, First Affiliated Hospital of Zhengzhou University, Zhengzhou, China

**Keywords:** deep vein thrombosis, nomogram, prediction model, risk assessment, sepsis, venous thromboembolism

## Abstract

**Background:**

Patients with sepsis are at high risk for venous thromboembolism (VTE). However, existing assessment tools designed for general medical or surgical populations may inadequately capture the specific risk profile of septic patients. This study aimed to identify independent predictors of VTE in sepsis and develop a sepsis-specific prediction model.

**Methods:**

In this retrospective cohort study, adult patients with sepsis diagnosed at a single center (2015–2024) were enrolled. Candidate predictors and treatments were restricted to data available within the first 24 h of diagnosis. After exclusions, 157 VTE cases and 703 matched non-VTE controls formed the prediction cohort, which was split into training (*n* = 602) and validation (*n* = 258) sets. The Synthetic Minority Oversampling Technique was applied to the training set. LASSO regression followed by multivariable logistic regression identified independent factors for the nomogram. Model performance was assessed via discrimination, calibration, and decision curve analysis and compared with the Padua and Caprini scores in the validation cohort.

**Results:**

Among 3,228 sepsis patients, 157 (4.86%) developed VTE. Multivariable analysis identified four independent predictors: surgery within 3 months, central venous catheterization within 24 h, history of VTE ≥1 year prior, and absence of early anticoagulant therapy (all *P* < 0.001). The resulting nomogram showed excellent discrimination (AUC: 0.905, 95% CI: 0.875–0.935 in training; 0.909, 95% CI: 0.867–0.951 in validation) and good calibration. Decision curve analysis supported its clinical utility. In the validation cohort, the nomogram significantly outperformed the Padua (AUC: 0.712) and Caprini (AUC: 0.738) scores, demonstrating superior incremental predictive value (*P* < 0.001 for net reclassification improvement and integrated discrimination improvement).

**Conclusions:**

A sepsis-specific nomogram incorporating recent surgery, early catheterization, previous VTE, and absence of early anticoagulation demonstrated good discrimination and calibration. It outperformed the Padua and Caprini tools in internal validation. External validation is required before clinical implementation.

## Introduction

Venous thromboembolism (VTE) refers to a disease characterized by abnormal coagulation of red blood cells, platelets, fibrinogen (FB), and other components within the deep venous lumen, leading to impaired blood flow in the limbs. It includes deep vein thrombosis (DVT) and pulmonary embolism (PE). Incidence rates for lower-extremity DVT range from 88 to 112 per 100,000 person-years and increase with age. Rates of recurrent VTE range from 20% to 36% during the 10 years after an initial event (1). VTE is a significant contributor to the global disease burden. Patients with VTE often face substantial economic costs and the risk of bleeding associated with anticoagulation therapy. In addition, these patients may experience long-term chronic complications, such as chronic lower limb edema, cutaneous malnutrition of the lower limbs, and even ulceration (2).

For a century, researchers have identified vascular endothelial damage, blood flow stasis, and hypercoagulability of the blood as the main factors contributing to thrombus formation, collectively termed Virchow's triad. Over the last decade, an increasing body of evidence suggests that inflammation may be a major contributor to the pathophysiology of VTE by enhancing hypercoagulability and increasing endothelial injury. During the inflammatory response, the activation of endothelial cells, platelets, and leukocytes triggers inflammation and microparticle formation, which in turn activates the coagulation system via the induction of tissue factor (TF), potentially leading to the hypercoagulable state described in Virchow's triad. Both the formation and resolution of thrombi are associated with a series of inflammatory cascades (3). In approximately 15% of thrombus samples obtained during pulmonary endarterectomy for PE, moderate-to-severe inflammation was detected (4).

Sepsis is a systemic inflammatory response syndrome (SIRS) caused by infection, which often induces organ dysfunction and can lead to patient death (5, 6). During systemic inflammatory response activity, endothelial injury and TF expression can trigger coagulation, leading to the activation and consumption of coagulation factors and platelets. This is accompanied by impaired fibrinolysis, disruption of the endothelial barrier, and loss of physiological antithrombotic factors (such as thrombomodulin), thereby increasing the risk of VTE (7, 8). In sepsis, there is a significant interplay between inflammation and coagulation, characterized primarily by the procoagulant properties of the endothelium. For example, the expression of TF and prethrombotic factors such as von Willebrand factor (vWF) (9) can activate interactions with platelets (10), the release of procoagulant microparticles, the downregulation of tissue factor pathway inhibitor (TFPI) (11) and the protein C/S anticoagulant system (12), and the inhibition of fibrinolysis (11). Patients with sepsis are highly susceptible to disseminated intravascular coagulation (DIC), which in turn can exacerbate the occurrence and progression of inflammation. In addition, while the coagulation response can block the invasion of pathogens, some bacteria have adapted to this response and can instead exploit the coagulation process to evade immune attacks (13).

In recent years, the incidence of sepsis has significantly increased due to factors such as population aging, the emergence of drug-resistant bacteria, and the widespread use of immunosuppressants and corticosteroids. The pathogens that cause sepsis are diverse, and include fungi, parasites, viruses, and bacteria (6, 14). Patients with sepsis often experience dysfunction of vital organs. The treatment of these patients frequently involves the use of mechanical ventilation, sedative agents, and invasive procedures, which can additionally promote the formation of VTE. These factors also readily induce severe complications such as systemic hypoxia, hemodynamic instability, and dysfunction of vital organs, ultimately leading to an increased mortality rate in sepsis patients (15, 16).

Several VTE risk assessment tools, including the Padua Prediction Score, Caprini Risk Assessment Model, and IMPROVE score, have been widely used in clinical practice for general medical and surgical populations (17). However, these models have notable limitations when applied to septic patients. First, they were primarily developed and validated in non-septic populations and lack sepsis-specific features such as infection site, sepsis-related severity scores [Sequential Organ Failure Assessment (SOFA), Glasgow Coma Scale (GCS)], and inflammatory markers (18). Second, the pathophysiology of VTE in sepsis is distinctly different from that in general medical patients, as sepsis involves a unique interplay between systemic inflammation and coagulation activation that existing models do not adequately capture. A systematic review and meta-analysis identified sepsis as an intensive care unit (ICU)-specific factor with high certainty of association with increased VTE risk (adjusted odds ratio, 1.41; 95% CI, 1.12–1.78) (19), highlighting the need for sepsis-specific risk stratification. Third, conventional risk stratification tools may inadequately capture the complex, non-linear interactions among clinical variables in septic patients (17). Therefore, a prediction model specifically designed for sepsis patients, incorporating sepsis-related variables, may provide additional clinical value beyond existing generic VTE risk assessment tools.

In clinical practice, if a scoring model is used to assess the risk of VTE in patients with sepsis, it could guide various potential thromboprophylaxis strategies in the early stage. Currently, there is no effective prediction model. We hope that through our research, we can explore the risk factors for VTE during sepsis and subsequently establish a VTE risk prediction model. These findings provide a basis for early prevention and intervention in clinical practice, reduce the incidence of VTE in patients with sepsis, and improve the prognosis of these patients.

This study aimed to establish a VTE risk prediction model for patients with sepsis based on their general condition, laboratory indicators, comorbidities, and ICU scoring systems. This study further evaluated the reliability of this prediction model to provide a screening tool for the early identification of adverse VTE outcomes in patients with sepsis.

## Methods

### Data sources

Clinical data from patients who were diagnosed with sepsis at our center from 2015 to 2024 were collected. According to the inclusion and exclusion criteria, a total of 3,228 patients diagnosed with sepsis were ultimately selected. The inclusion criteria for patients were as follows: (1) met the diagnostic criteria for sepsis in the Chinese Emergency Treatment Guidelines for Sepsis/Septic Shock (2018); and (2) VTE patients were defined as those who developed new-onset venous thrombosis confirmed by vascular ultrasound or contrast-enhanced CT during the diagnosis and treatment of sepsis. The exclusion criteria were as follows: (1) pregnancy, puerperium, or under 18 years of age; and (2) incomplete clinical data. This study was approved by the Ethics Committee of the Huaihe Hospital of Henan University (approval number 2025053). Given the retrospective nature of this study, the ethics committee consent the requirement for informed, and all patient data were anonymized prior to analysis. The retrospective data were used in accordance with strict privacy protection and anonymization principles.

A retrospective analysis of clinical data from patients diagnosed with sepsis at our center from 2015 to 2024, and patients were divided into VTE and non-VTE groups. The following basic information was collected for each patient: (1) demographic information, including age, gender, body mass index (BMI), smoking status, and alcohol consumption; (2) underlying diseases and comorbidities, such as a history of diabetes, hypertension, stroke, chronic lung disease (chronic obstructive pulmonary disease and/or asthma), kidney disease, malignancy, autoimmune disease, surgery within the 3 months prior to diagnosis, valvular heart disease, and arrhythmia; (3) treatments received during hospitalization, including whether central venous catheterization was performed and the site of catheter placement, whether corticosteroids were used, and whether the patient was bedridden for ≥3 days; and (4) laboratory test results: for all participants, candidate predictor variables were defined using information available within 24 h after the diagnosis of sepsis and before the occurrence of VTE—for patients who subsequently developed VTE, only measurements obtained before VTE diagnosis were considered, whereas for patients without VTE, measurements obtained within the same 24 h window after sepsis diagnosis were considered, with laboratory parameters including C-reactive protein (CRP), white blood cell (WBC) count, red blood cell (RBC) count, platelet (PLT) count, mean platelet volume (MPV), activated partial thromboplastin time (APTT), prothrombin time (PT), FIB, D-dimer, and triglyceride (TG) levels, alongside blood culture results and sites of infection during hospitalization; and (5) sepsis-related severity scores, including the SOFA, GCS, bilirubin levels, and creatinine levels.

### Statistical analysis

Patients were divided into a positive group (VTE group, *n* = 157) and a negative group (non-VTE group, *n* = 3,071) based on whether they developed VTE. To develop a clinical prediction model, at least 5–10 events are required per predictor variable. From the 3,071 non-VTE patients, 703 were selected using simple random sampling with a fixed random seed (set.seed = 2,024) to serve as the negative control group. The ratio of approximately 1:4.5 between VTE and non-VTE cases was prespecified to maintain sufficient non-events for model stability while limiting the computational burden associated with extreme class imbalance during model development. The selected non-VTE patients were not preferentially selected based on any outcome-related characteristics. Patients with incomplete data on key variables (defined as missing >20% of the candidate predictors) were excluded prior to sampling. The specific variables with missing values and their respective missing proportions are summarized in Supplementary Table S1.

All model development procedures, including LASSO feature selection, were performed exclusively in the training cohort (*n* = 602). The validation cohort (*n* = 258) was kept completely independent and was not used for feature selection, oversampling, model fitting, or parameter tuning.

Given the imbalance between positive and negative samples (VTE incidence 4.86%), the Synthetic Minority Oversampling Technique (SMOTE) was applied exclusively to the training set to achieve a 1:1 ratio between VTE and non-VTE cases. SMOTE creates synthetic minority-class samples by interpolating between existing minority-class instances. The validation set was not oversampled and retained its original class distribution to ensure an unbiased evaluation of model performance. To assess the potential impact of oversampling on model calibration, we performed a sensitivity analysis comparing model performance with and without SMOTE (Supplementary Table S2).

To benchmark the performance of our proposed nomogram against established VTE risk assessment tools, we applied the Padua Prediction Score and the Caprini Risk Assessment Model to the validation cohort. The Padua score was calculated based on 11 risk factors, including active cancer, previous VTE, reduced mobility, thrombophilic conditions, recent trauma or surgery, age ≥70 years, heart or respiratory failure, acute myocardial infarction or ischemic stroke, acute infection or rheumatologic disease, obesity, and ongoing hormonal treatment. The Caprini score was calculated using risk factors including age, BMI, medical history, surgery type, and laboratory abnormalities. Both scores were applied using previously published criteria. The IMPROVE VTE score could not be calculated reliably because several required variables were not routinely recorded in the retrospective database. Therefore, the comparison was limited to the Padua and Caprini scores. The AUC, sensitivity, specificity, and calibration of these scores in the validation cohort were compared with those of our nomogram model. In addition, net reclassification improvement (NRI) and integrated discrimination improvement (IDI) were calculated to quantify the incremental value of our sepsis-specific model over the Padua and Caprini scores.

Continuous variables with a normal distribution are presented as the means ± standard deviations (x¯ ± s), and comparisons between groups were performed using the independent samples *t*-test. For skewed distributions, data are presented as medians and interquartile ranges [M, (Q25, Q75)], and the Mann–Whitney *U*-test was used for comparisons. Categorical variables are expressed as percentages [(*n* (%)], and comparisons were made using the Pearson chi-square test or Fisher's exact test. Variables with statistically significant differences in the univariate analysis were included in the Lasso logistic regression model to select characteristic variables. Multivariate logistic regression analysis was then performed on the selected variables to identify independent risk factors. Based on the results of the multivariate logistic regression analysis, a nomogram was constructed using R software (version 4.3.1). Moreover, calibration curves, receiver operating characteristic (ROC) curves, and decision curves were generated to evaluate the calibration, discrimination, and clinical utility of the prediction model. A *P*-value of <0.05 was considered to indicate statistical significance. Furthermore, patients were randomly divided into a training set (*n* = 602) and a validation set (*n* = 258) in a 7:3 ratio.

## Results

### Baseline characteristics of patients with sepsis

A retrospective analysis was conducted on 3,228 patients diagnosed with sepsis, of whom 157 were in the VTE group and 3,071 were in the non-VTE group, corresponding to an incidence rate of 4.86%. The types of VTE observed included DVT (*n* = 93, 59.3%), PE (*n* = 2, 1.3%), venous catheter-related thrombosis (*n* = 40, 25.5%), hepatic/portal vein thrombosis (*n* = 16, 10.2%), brachial vein thrombosis (*n* = 1, 0.6%), inferior vena cava thrombosis (*n* = 3, 1.9%), superior mesenteric vein thrombosis (*n* = 1, 0.6%), and intracranial vein thrombosis (*n* = 1, 0.6%). The following baseline characteristics were significantly different between the two groups: history of surgery within 3 months prior to the diagnosis of sepsis, history of central venous catheterization during treatment, bedridden status for ≥3 days, and history of prior VTE (defined as any previous VTE episode documented in the patient's medical records, regardless of time since the event). During the treatment of sepsis, comparisons between the two groups revealed that the site of infection, hemoglobin, PT, APTT, D-dimer, bilirubin, creatinine, GCS, SOFA, use of corticosteroids before thrombus formation, and absence of anticoagulant therapy were statistically significant (all *P* < 0.05). The other indicators were not significantly different (Table 1).

**Table 1:** Baseline characteristics of patients with sepsis.

Variables	VTE (*n* = 157)	Non-VTE (*n* = 703)	T/Z/X2	*P*
Sex, *n* (%)				
Female	63 (40.1)	314 (44.7)	X2	0.3
Male	94 (59.9)	389 (55.3)	1.074	
Age (x ± s)	66.00 (54.00, 71.50)	66.00 (54.00, 75.00)	Non-normal distribution, *Z* = −1.166	0.243
Site of infection, *n* (%)	0: Venous catheter, 18 (11.5)	0: Venous catheter, 10 (1.4)	X2	0.000
	1: Pulmonary infection, 87 (55.4)	1: Pulmonary infection, 336 (47.8)	52.123	
	2: Abdominal infection, 15 (9.6)	2: Abdominal infection, 82 (11.7)		
	3: Liver and biliary infection, 14 (8.9)	3: Liver and biliary infection, 89 (12.7)		
	4: Urinary tract infection, 6 (3.8)	4: Urinary tract infection, 65 (9.2)		
	5: Bloodstream infection, 10 (6.4)	5: Bloodstream infection, 88 (12.5)		
	6: Other infections (skin, brain, etc.), 7 (4.5)	6: Other infections (skin, brain, etc.), 33 (4.7)		
Etiology, *n* (%)	0: Fungus, 14 (8.9)	0: Fungus, 71 (10.1)	Fisher's exact test	0.731
	1: Enteric bacilli, 65 (41.4)	1: Enteric bacilli, 278 (39.5)	X2	
	2: Non-fermentative bacteria/non-fermentative enterobacterium, 18 (11.5)	2: Non-fermentative bacteria/non-fermentative enterobacterium, 102 (14.5)	2.754	
	3: *Staphylococcus*, 36 (22.9)	3: *Staphylococcus*: 130 (18.5)		
	4: *Streptococcus*/*Enterococcus* genus, 24 (15.3)	4: *Streptococcus*/*Enterococcus* genus, 120 (17.1)		
	5: Other, 0 (0)	5: Other, 2 (0.3)		
Basic diseases, *n* (%)	0: Non, 51 (32.5)	0: Non, 282 (40.1)	X2	0.000
	1: Malignant tumor, 12 (7.6)	1: Malignant tumor, 42 (6.0)	36.726	
	2: Cerebral infarction/cerebral hemorrhage, 13 (8.3)	2: Cerebral infarction/cerebral hemorrhage, 110 (15.6)		
	3: Hypertension, 55 (35.0)	3: Hypertension, 137 (19.5)		
	4: Coronary heart disease, 2 (1.3)	4: Coronary heart disease, 40 (5.7)		
	5: Hepatitis B, 6 (3.8)	5: Hepatitis B, 27 (3.8)		
	6: Diabetes, 5 (3.2)	6: Diabetes, 43 (6.1)		
	7: Other, 13 (8.3)	7: Other, 22 (3.1)		
History of surgery, *n* (%)	N, 81 (51.6)	N, 492 (70.0)	X2	0.000
	Y, 76 (48.4)	Y, 211 (30.0)	19.527	
Intravenous catheterization, *n* (%)	N, 72 (45.9)	N, 609 (86.6)	X2	0.000
	Y, 85 (54.1)	Y, 94 (13.4)	129.423	
Bedridden for at least 3 days, *n* (%)	N, 111 (70.7)	N, 552 (78.5)	X2	0.035
	Y, 46 (29.3)	Y, 151 (21.5)	4.444	
VTE history, *n* (%)	N, 140 (89.2)	N, 700 (99.6)	X2	0.000
	Y, 17 (10.8)	Y, 3 (0.4)	56.632	
Blood pressure during treatment, *n* (%)	0: Hypotension, 10 (6.4)	0: Hypotension, 32 (4.6)	X2	0.285
	1: Normal, 93 (59.2)	1: Normal, 480 (68.3)	5.027	
	2: Hypertension grade 1, 29 (18.5)	2: Hypertension grade 1,96 (13.7)		
	3: Hypertension grade 2, 13 (8.3)	3: Hypertension grade 2, 49 (7.0)		
	4: Hypertension grade 3, 12 (7.6)	4: Hypertension grade 3, 46 (6.5)		
Smoking history, *n* (%)	N, 120 (76.4)	N, 547 (77.8)	X2	0.709
	Y, 37 (23.6)	Y, 156 (22.2)	0.140	
Drinking history, *n* (%)	N, 133 (84.7)	N, 619 (88.1)	X2	0.254
	Y, 24 (15.3)	Y, 84 (11.9)	1.302	
Using glucocorticoids, *n* (%)	N, 86 (54.8)	N, 496 (70.6)	X2	0.000
	Y, 71 (45.2)	Y, 207 (29.4)	14.604	
Anticoagulant therapy, *n* (%)	N, 131 (83.4)	N, 131 (18.6)	X2	0.000
	Y, 26 (16.6)	Y, 572 (81.4)	254.43	
CRP [M (Q25, Q75)]	78.61 (31.60, 138.125)	63.3 (24.9, 118.3)	Non-normal distribution, *Z* = −2.141	0.032
WBC [M (Q25, Q75)]	8.97 (5.74, 14.55)	8.80 (5.82, 13.10)	Non-normal distribution, *Z* = −0.573	0.566
Hb [M (Q25, Q75)]	96 (83, 115.50)	109 (92, 123)	Non-normal distribution, *Z* = −4.63	0.000
PLT [M (Q25, Q75)]	157 (99.5, 242.5)	176 (110, 241)	Non-normal distribution, *Z* = −0.622	0.534
Mean platelet volume [M (Q25, Q75)]	10.4 (10.0, 11.2)	10.6 (9.9, 11.4)	Non-normal distribution, *Z* = −1.47	0.142
PT [M (Q25, Q75)]	14.1 (13.1, 15.9)	13.7 (12.8, 14.8)	Non-normal distribution, *Z* = −2.826	0.005
APTT [M (Q25, Q75)]	40.75 (35.8, 46.9)	37.8 (34.3, 42.1)	Non-normal distribution, *Z* = −4.172	0.000
DD [M (Q25, Q75)]	2,280 (1,222.5, 3,735)	1,390 (821, 2,284)	Non-normal distribution, *Z* = −5.385	0.000
Fib [M (Q25, Q75)]	407.5 (305.5, 500.5)	416 (341, 512)	Non-normal distribution, *Z* = −1.196	0.232
Bilirubin [M (Q25, Q75)]	11.2 (7.43, 17.25)	13.95 (8.91, 25.90)	Non-normal distribution, *Z* = −4.036	0.000
Creatinine [M (Q25, Q75)]	67 (47, 187)	64 (50, 91)	Non-normal distribution, *Z* = −2.321	0.02
Sofa (x ± s)	5 (3, 7)	3 (2, 6)	Non-normal distribution, *Z* = −4.949	0.000
GCS [M (Q25, Q75)]	13 (6, 14)	15 (12, 15)	Non-normal distribution, *Z* = −7.991	0.000
Site of thrombus (VTE), *n* (%)	DVT, 93 (59.3)			
	PE, 2 (1.3)			
	Venous catheterization, 40 (25.5)			
	Hepatic/portal vein, 16 (10.2)			
	Brachial vein, 1 (0.6)			
	Inferior vena cava, 3 (1.9)			
	Superior mesenteric vein, 1 (0.6)			
	Intracranial vein, 1 (0.6)			

### Risk prediction model for VTE in patients with sepsis

In the training cohort, LASSO regression was employed for feature selection. Given the imbalance between positive and negative samples in the training set, SMOTE oversampling of the minority class was performed to achieve a 1:1 balance in the training set only. In addition, continuous variables were normalized. A total of 16 factors with statistical significance were initially selected and subjected to Lasso logistic regression analysis (Figure 1). Tenfold cross-validation was performed, and the lambda value that yielded the lowest test mean squared error (MSE) was determined. As shown in the mean squared error and coefficient convergence plot, the model achieves the minimum MSE at the position indicated by the red line when 14 data items are selected. At this point, the lambda value was set to 0.0021, corresponding to the selection of 14 factors that minimized the MSE. The 14 potential predictive factors for VTE in patients with sepsis identified by the model are as follows: site of infection, history of surgery, intravenous catheterization, bedridden for at least 3 days, VTE history, use of glucocorticoids, CRP, PT, APTT, D-dimer (DD), bilirubin, creatinine, SOFA score, and GCS score. With VTE as the dependent variable, multivariate logistic regression was subsequently performed. The results revealed that a history of surgery within 3 months prior to the diagnosis of sepsis, a history of central venous catheterization during treatment, a history of VTE for ≥1 year, and the absence of anticoagulant therapy were independent risk factors for VTE in patients with sepsis (all *P* < 0.001, Table 2). A nomogram prediction model was constructed based on these four variables (Figure 2). The scores assigned to each variable in the nomogram were as follows: surgery history (0 = 0 points, 1 = 29 points), central venous catheterization (0 = 0 points, 1 = 47 points), VTE history (0 = 0 points, 1 = 100 points), and absence of anticoagulant therapy (0 = 0 points, 1 = 82 points).

**Figure 1:**
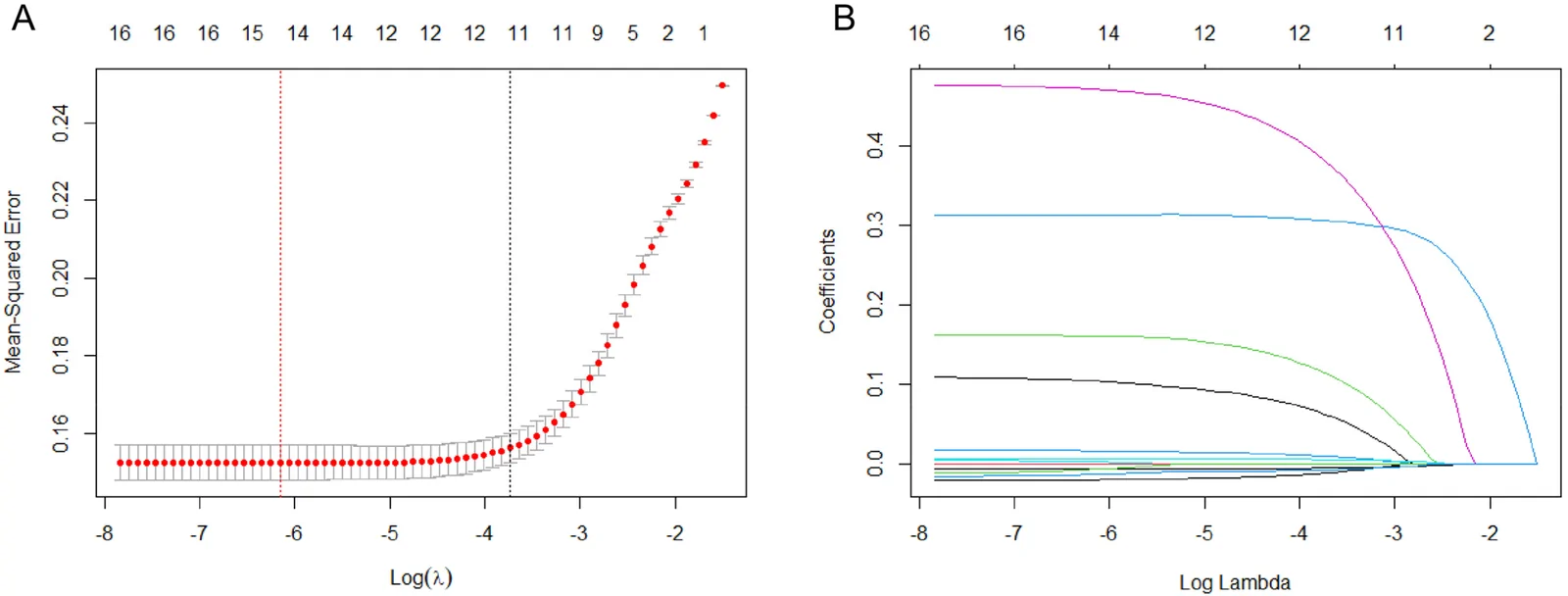
LASSO regression analysis for selecting risk factors for VTE in patients with sepsis. **(A)** Curve showing the relationship between the number of predictors and log(*λ*); the *y*-axis represents the deviance. The optimal *λ* value was selected via 10-fold cross-validation based on the minimum mean squared error. **(B)** Coefficient trajectories of each predictor as the penalty parameter *λ* varies.

**Table 2:** Multivariate logistic regression analysis of risk factors for VTE in patients with sepsis.

Variables	Wald	*P*	Odds ratio (OR)	95% CI	
				Lower	Upper
History of surgery	17.565	0	2.852	1.747	4.656
Central venous catheterization	48.859	0	5.64	3.472	9.161
VTE history	20.536	0	39.053	8.003	190.568
Absence of anticoagulant therapy	130.466	0	20.457	12.187	34.337

**Figure 2:**
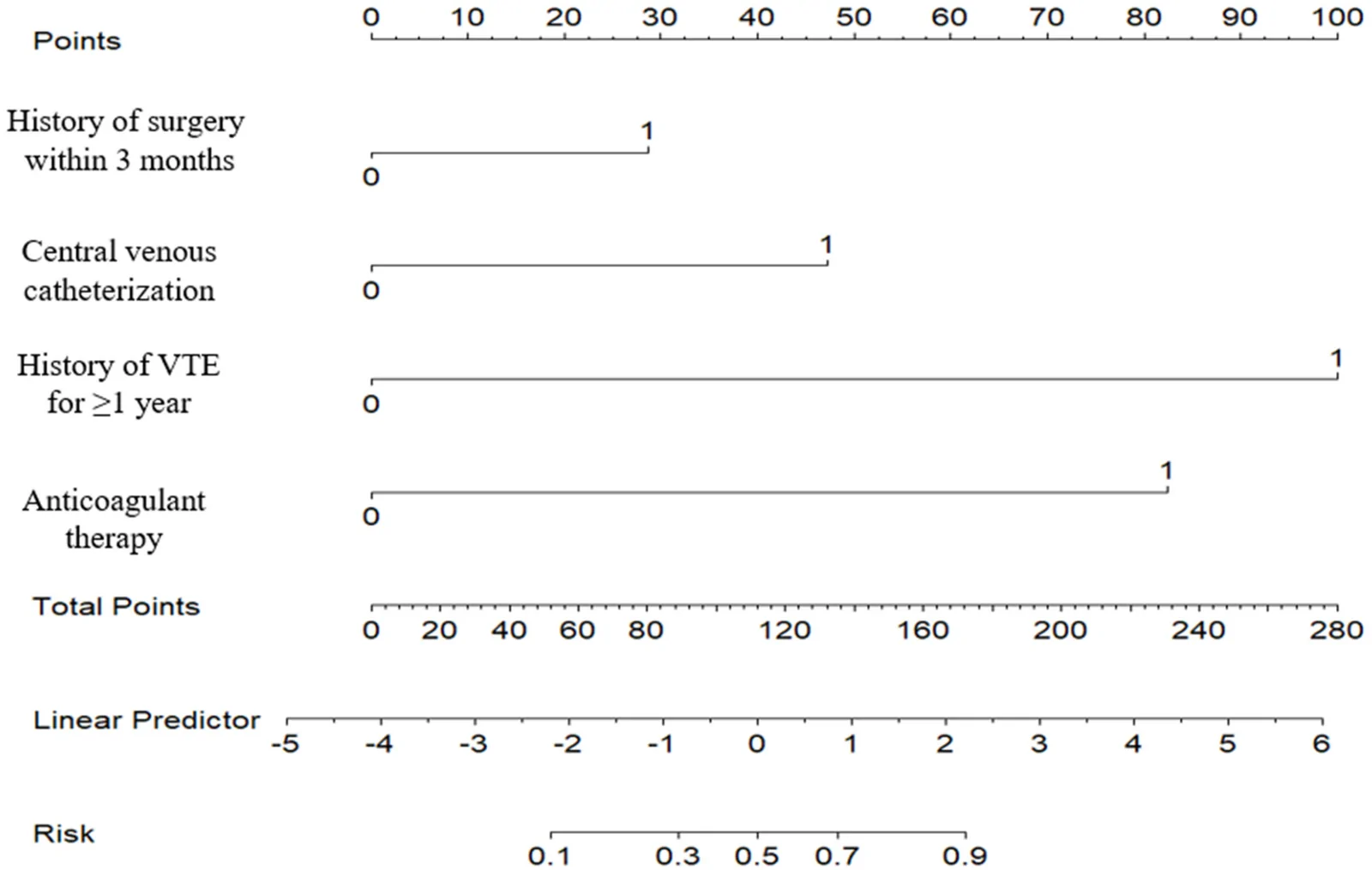
Nomogram for predicting the risk of VTE in patients with sepsis. The nomogram was constructed based on four variables: history of surgery within 3 months prior to sepsis diagnosis, history of central venous catheterization during treatment, history of VTE for ≥1 year, and absence of anticoagulant therapy. Points are assigned to each variable, and the total score corresponds to the predicted probability of VTE.

A SHapley Additive exPlanations (SHAP) analysis was performed using the entire dataset for modeling. The features were ranked by importance, from highest to lowest, as follows: absence of anticoagulant therapy, history of central venous catheterization during treatment, history of surgery within 3 months prior to the diagnosis of sepsis, and history of VTE for ≥1 year. Among these factors, history of VTE had a significant effect on the outcome when it was present (value = 1), but it had minimal influence when it was absent (value = 0) (Figure 3).

**Figure 3:**
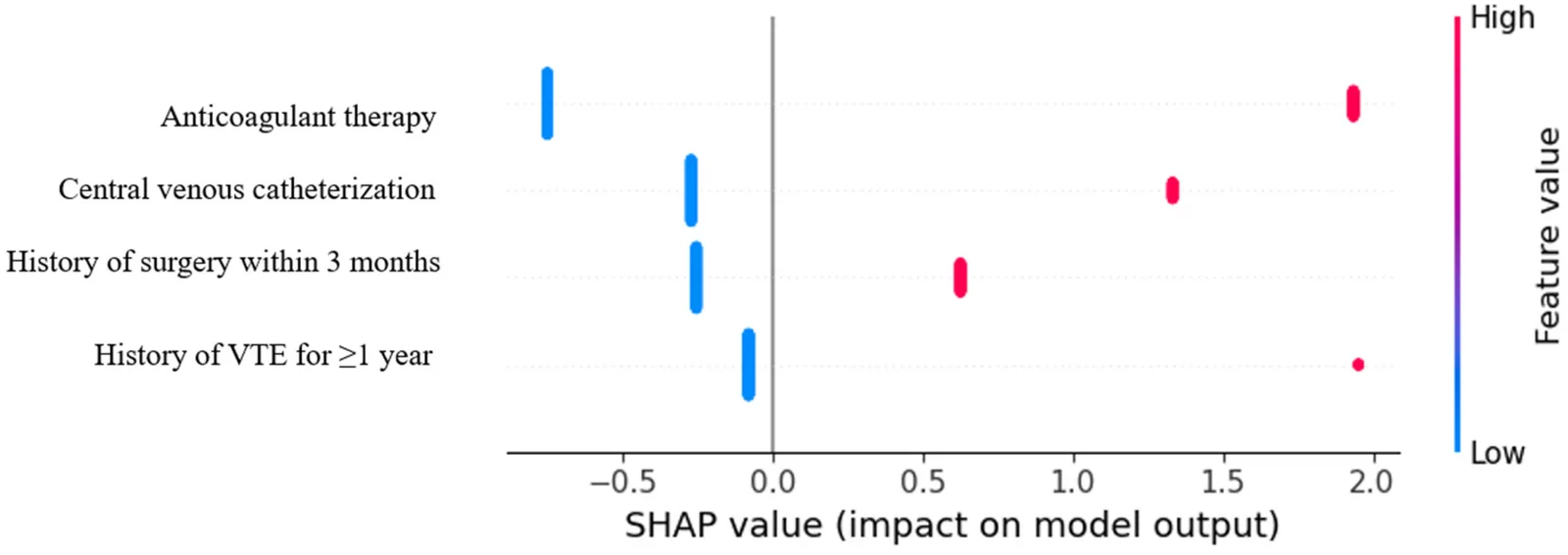
SHAP analysis of feature contributions. The features are ranked by importance from highest to lowest. Red indicates higher feature values, and blue indicates lower feature values. The *x*-axis represents the SHAP value, which quantifies the contribution of each feature to the predicted outcome.

### Evaluation of the discriminative, calibration, and clinical utility of the nomogram model

The performance of the model was evaluated using a confusion matrix. Figure 4A shows the confusion matrix for the training set, with an accuracy of 88.37%; Figure 4B shows the confusion matrix for the validation set, with an accuracy of 87.21%.

**Figure 4:**
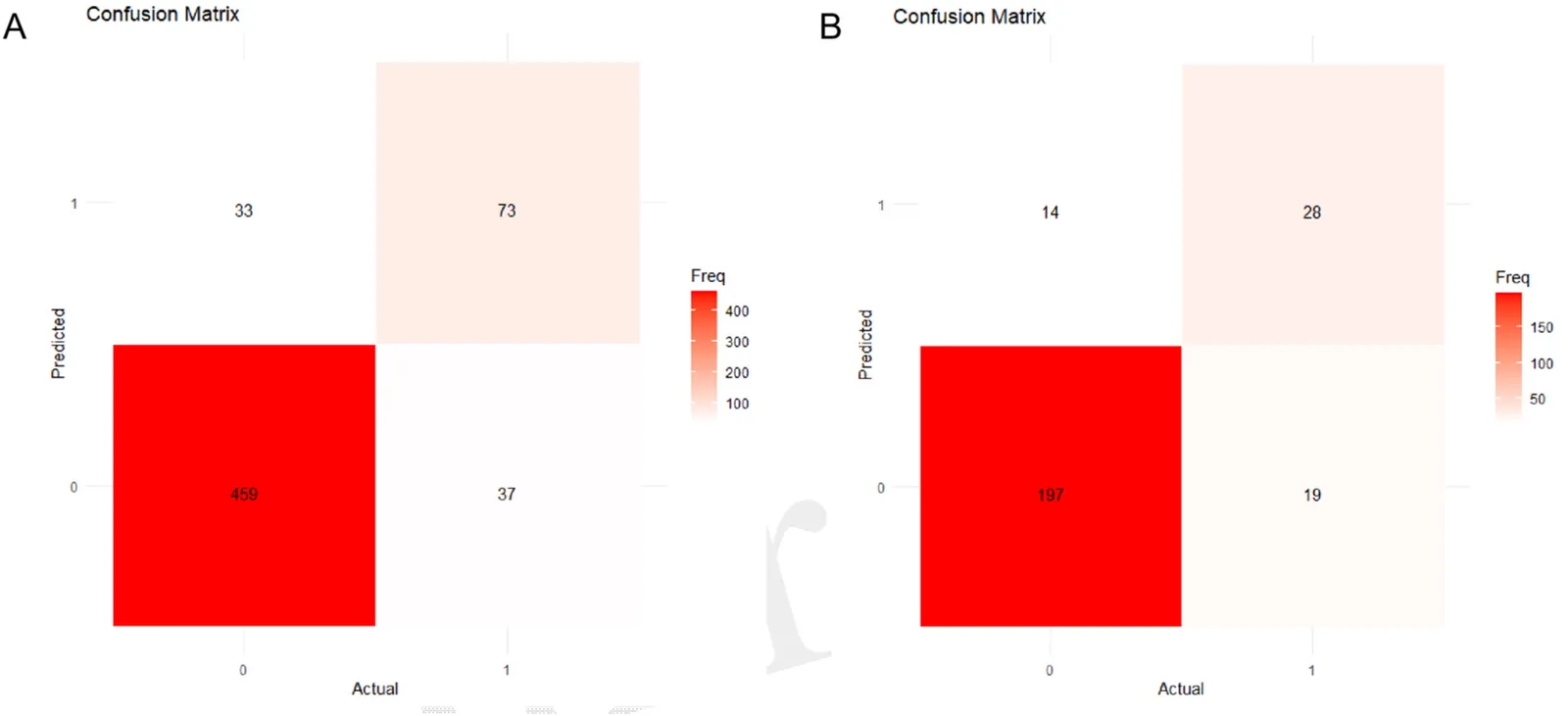
Confusion matrix of the nomogram prediction model. **(A)** Training set (accuracy: 88.37%). **(B)** Validation set (accuracy: 87.21%). TN, true negative; FP, false positive; FN, false negative; TP, true positive.

Figure 5A shows the ROC curves for the training set, which were constructed using three classification methods: the nomogram model (machine learning), GCS, and SOFA. The *x*-axis represents 1 − specificity, and the *y*-axis represents sensitivity. The ROC curve for the machine learning model had an area under the curve (AUC) of 0.905, with a 95% CI of 0.875–0.935, a sensitivity of 66.36%, and a specificity of 93.3%, indicating its ability to predict the risk of VTE occurrence. While the ROC curve for the GCS score had an AUC of 0.683, with a 95% CI of 0.637–0.730, the ROC curve for the SOFA score had an AUC of 0.628, with a 95% CI of 0.574–0.683.

**Figure 5:**
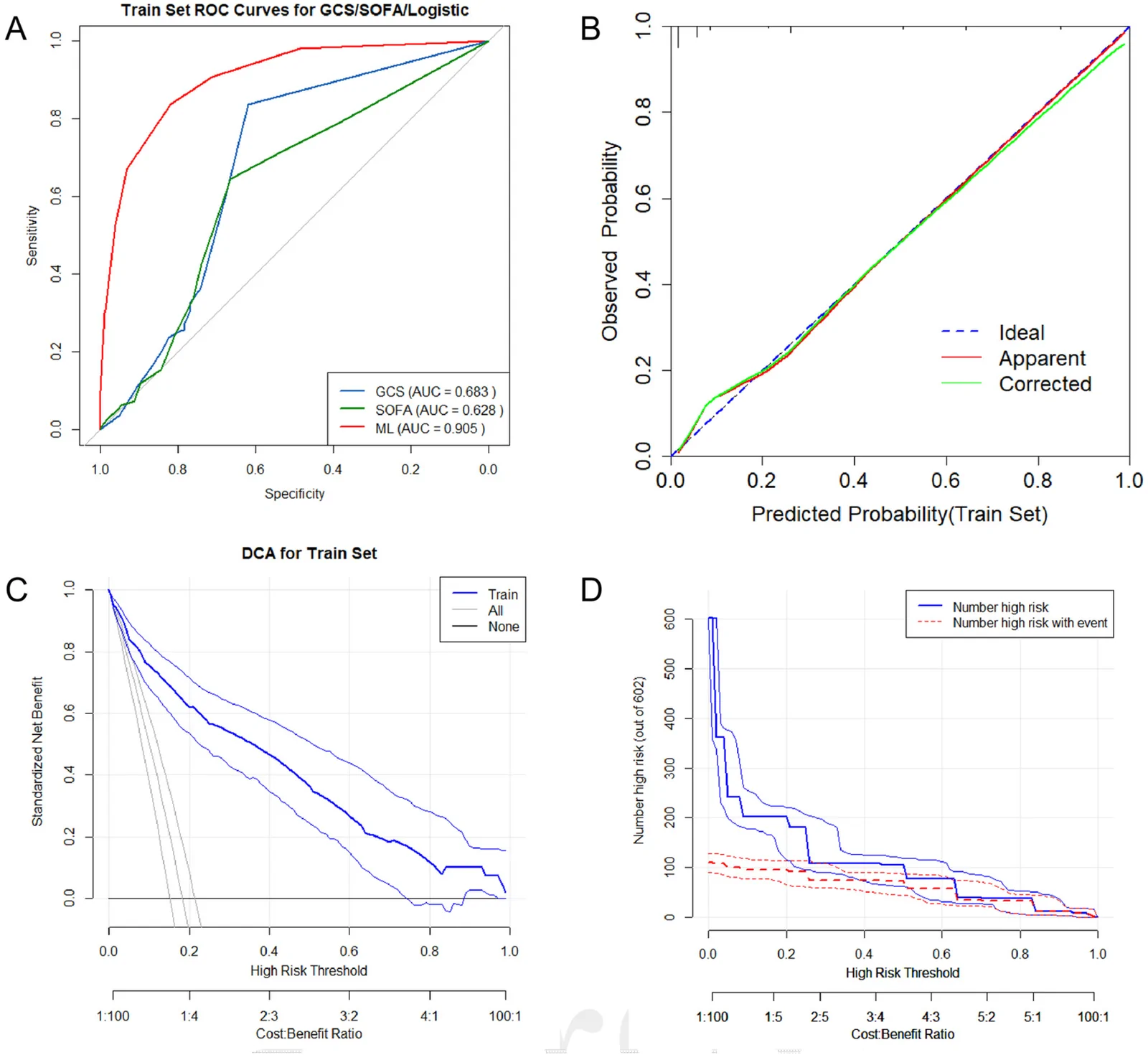
Performance evaluation of the nomogram prediction model for VTE risk in patients with sepsis (training set). **(A)** ROC curves comparing the nomogram model, GCS score, and SOFA score. **(B)** Calibration curve with bootstrap resampling (1,000 replications). **(C)** DCA including “treat all” and “treat none” reference lines. **(D)** CIC.

To benchmark our model against established VTE risk assessment tools in the same validation cohort, we calculated the Padua Prediction Score and Caprini Risk Assessment Model for each patient in the validation set. The Padua score achieved an AUC of 0.712 (95% CI: 0.641–0.783) with a sensitivity of 48.94% and specificity of 78.67%. The Caprini score achieved an AUC of 0.738 (95% CI: 0.669–0.807) with a sensitivity of 55.32% and specificity of 75.83%. In comparison, our nomogram model demonstrated superior discrimination with an AUC of 0.909 (95% CI: 0.867–0.951). The NRI of our model compared with the Padua score was 0.324 (95% CI: 0.187–0.461, *P* < 0.001) and compared with the Caprini score was 0.287 (95% CI: 0.152–0.422, *P* < 0.001). The IDI compared with the Padua score was 0.156 (95% CI: 0.098–0.214, *P* < 0.001) and compared with the Caprini score was 0.138 (95% CI: 0.082–0.194, *P* < 0.001). These results indicate that our sepsis-specific nomogram provides significant incremental predictive value over existing generic VTE risk assessment tools.

Using the bootstrap resampling method, a calibration curve was generated with 1,000 replications. Figure 5B shows the calibration curve for the training data. The term “Apparent” indicates the fit between the predicted and actual values, whereas “Corrected” represents the fit after calibration. Both lines are close to the ideal reference line (ideal), indicating good calibration and strong consistency between the predicted risk and actual incidence.

The decision curve analysis (DCA) (Figure 5C) indicates that our logistic regression model has a greater benefit within the medium probability range, with a trend toward the upper right, suggesting good performance. The DCA includes “treat all” and “treat none” reference lines, demonstrating that our model provides superior net benefit across a wide range of threshold probabilities compared with these default strategies. The clinical impact curve (CIC) (Figure 5D) demonstrates that the two lines are close when the probability threshold is greater than 0.4, indicating a good fit. The two thinner lines above and below the main curve represent the 95% confidence intervals, suggesting significant predictive value.

### Model validation

Patients were randomly divided into a training set (*n* = 602) and a validation set (*n* = 258) in a 7:3 ratio. As can be seen from Table 3, the *P*-values of all fields in the training and validation sets are greater than 0.05, showing no statistical difference (Table 3).

**Table 3:** Baseline characteristics of the training cohort and the validation cohort.

Variables	Training cohort (*n* = 602)	Validation cohort (*n* = 258)	T/Z/X2	*P*
	VTE 110, non-VTE 492	VTE 47, non-VTE 211		
Site of infection, *n* (%)	0: Venous catheter, 21 (3.5)	0: Venous catheter, 7 (2.7)	X2	0.227
	1: Pulmonary infection, 290 (48.2)	1: Pulmonary infection, 133 (51.6)	8.149	
	2: Abdominal infection, 76 (12.6)	2: Abdominal infection, 21 (8.1)		
	3: Liver and biliary infection, 67 (11.1)	3: Liver and biliary infection, 36 (14.0)		
	4: Urinary tract infection, 55 (9.1)	4: Urinary tract infection, 16 (6.2)		
	5: Bloodstream infection, 68 (11.3)	5: Bloodstream infection, 30 (11.6)		
	6: Other infections (skin, brain, etc.), 25 (4.2)	6: Other infections (skin, brain, etc.), 15 (5.8)		
Basic diseases, *n* (%)	0: Non, 238 (39.5)	0: Non, 95 (36.8)	X2	0.728
	1: Malignant tumor, 40 (6.6)	1: Malignant tumor, 14 (5.4)	4.44	
	2: Cerebral infarction/cerebral hemorrhage, 91 (15.1)	2: Cerebral infarction/cerebral hemorrhage, 32 (12.4)		
	3: Hypertension, 127 (21.1)	3: Hypertension, 65 (25.2)		
	4: Coronary heart disease, 26 (4.3)	4: Coronary heart disease, 16 (6.2)		
	5: Hepatitis B, 23 (3.8)	5: Hepatitis, B 10 (3.9)		
	6: Diabetes, 33 (5.5)	6: Diabetes, 15 (5.8)		
	7: Other, 24 (4.0)	7: Other, 11 (4.3)		
History of surgery, *n* (%)	N, 403 (66.9%)	N, 170 (65.9%)	X2	0.764
	Y, 199 (33.1%)	Y, 88 (34.1%)	0.09	
Intravenous catheterization, *n* (%)	N, 477 (79.2%)	N, 204 (79.1%)	X2	0.956
	Y, 125 (20.8%)	Y, 54 (20.9%)	0.003	
Bedridden for at least 3 days, *n* (%)	N, 475 (78.9%)	N, 188 (72.9%)	X2	0.054
	Y, 127 (21.1%)	Y, 70 (27.1%)	3.725	
VTE history, *n* (%)	N, 587 (97.5%)	N, 253 (98.1%)	X2	0.622
	Y, 15 (2.5%)	Y, 5 (1.9%)	0.244	
Using glucocorticoids, *n* (%)	N, 405 (67.3)	N, (68.6)	X2	0.703
	Y, 197 (32.7)	Y, (31.4)	0.146	
Anticoagulant therapy, *n* (%)	N, 418 (69.4)	N, 180 (69.8)	X2	0.923
	Y, 184 (30.6)	Y, 78 (30.2)	0.009	
CRP [M (Q25, Q75)]	63.3 (25.69, 119.28)	63.3 (24.9, 134.93)	Non-normal distribution, *Z* = −0.103	0.918
Hb [M (Q25, Q75)]	106.19 (SD = 22.53)	105.84 (SD = 23.71)	Normal distribution, *T* = 0.206	0.836
PT [M (Q25, Q75)]	13.7 (12.9, 14.9)	13.7 (13, 15.1)	Non-normal distribution, *Z* = −0.897	0.37
APTT [M (Q25, Q75)]	38.1 (34.68, 43.1)	37.8 (34.18, 42.7)	Non-normal distribution, *Z* = −1.048	0.295
DD [M (Q25, Q75)]	1,390 (868.75, 2,571.5)	1,390 (1,077.25,3, 674.25)	Non-normal distribution, *Z* = −1.799	0.072
Bilirubin [M (Q25, Q75)]	12.7 (8.17, 24.27)	14.46 (9.39, 25.4)	Non-normal distribution, *Z* = −1.829	0.067
Creatinine [M (Q25, Q75)]	64 (49, 100)	67 (48,101.25)	Non-normal distribution, *Z* = −0.265	0.791
Sofa (x ± s)	3 (2, 6)	4 (2, 7)	Non-normal distribution, *Z* = −0.64	0.522
GCS [M (Q25, Q75)]	15 (11, 15)	15 (9, 15)	Non-normal distribution, *Z* = −0.81	0.418

In the validation cohort, the confusion matrix showed an accuracy of 87.21%. The ROC curve (Figure 6A) demonstrated that the machine learning model had an AUC of 0.909, with a 95% CI of 0.867–0.951, a sensitivity of 59.57%, and a specificity of 93.03%. The ROC curve for the GCS score had an AUC of 0.695, with a 95% CI of 0.624–0.766; the ROC curve for the SOFA score had an AUC of 0.612, with a 95% CI of 0.527–0.697. The calibration curve (Figure 6B) indicated that the model fit was not as good as that in the training set but was still satisfactory overall. Both the DCA (Figure 6C) and the CIC (Figure 6D) suggested that the model had good predictive significance.

**Figure 6:**
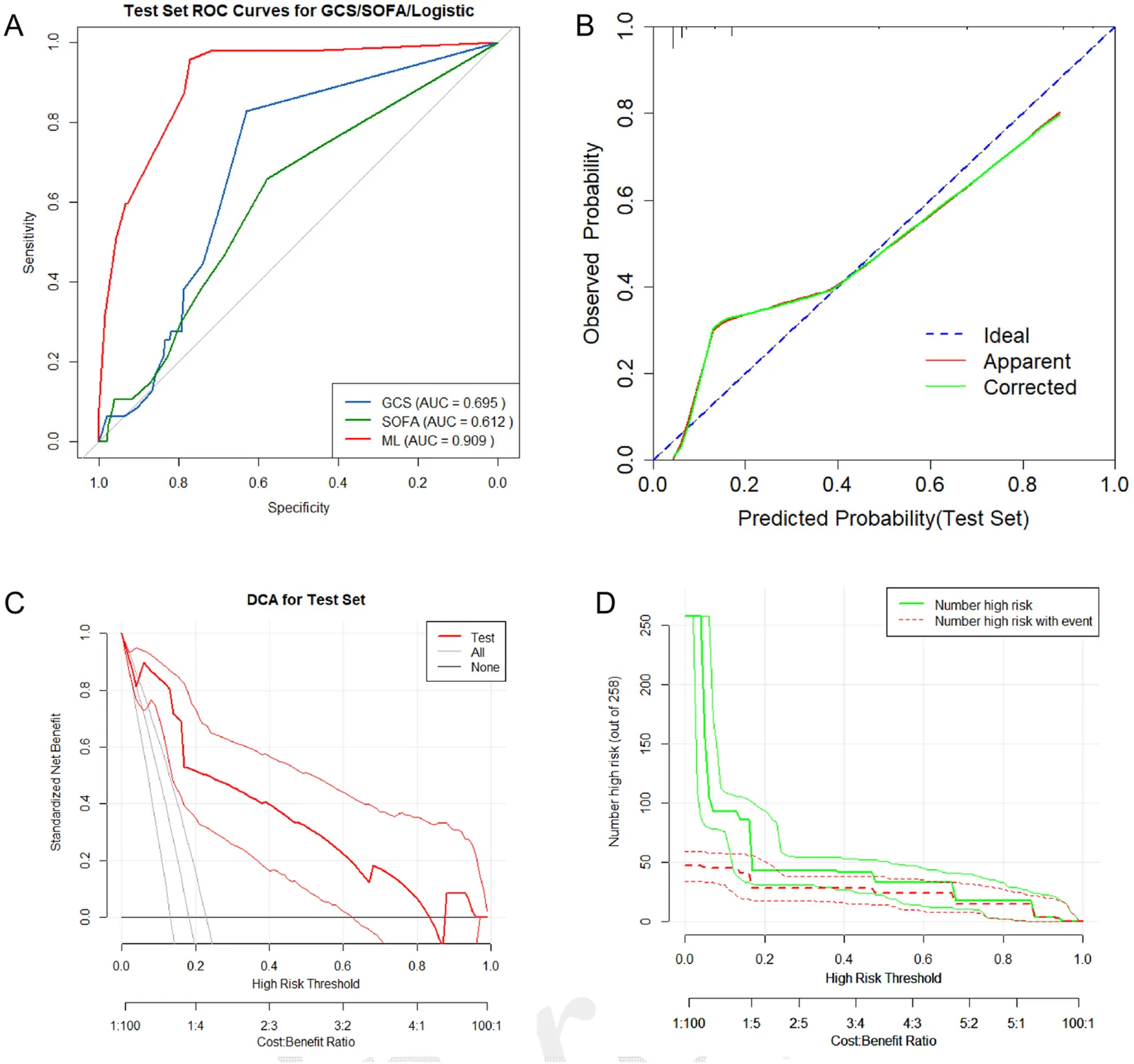
Performance evaluation of the nomogram prediction model in the validation cohort. **(A)** ROC curves comparing the nomogram model, GCS score, and SOFA score. **(B)** Calibration curve. **(C)** Decision curve analysis (DCA). **(D)** CIC.

### App for predicting VTE in sepsis patients

We developed an app to predict VTE in sepsis patients using a nomogram model and machine learning (Figure 7).

**Figure 7:**
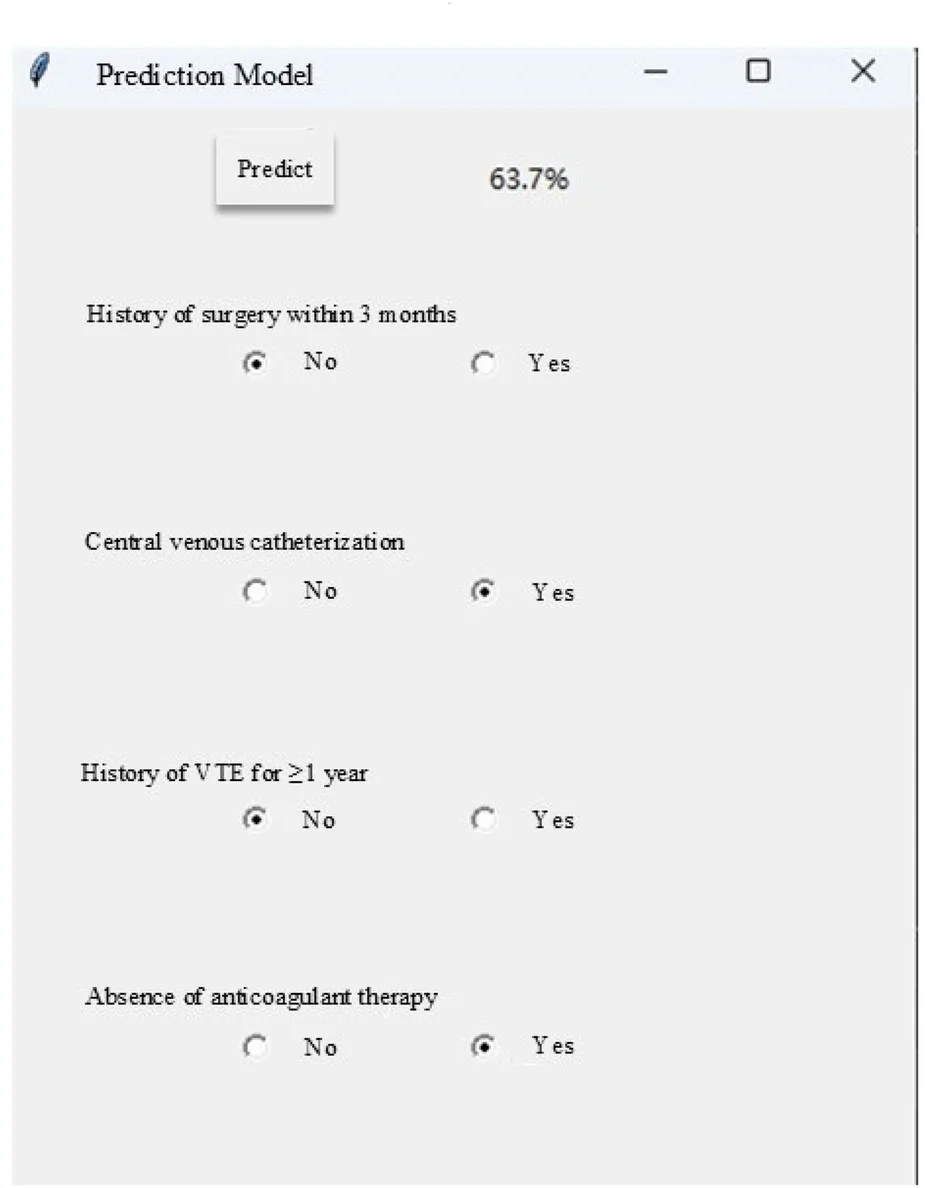
User interface of the mobile application for predicting VTE in sepsis patients based on the nomogram model.

## Discussion

Our study identified independent risk factors for venous thrombosis in patients with sepsis using Lasso logistic regression and developed a nomogram prediction model. The results revealed that a history of surgery within 3 months, a history of central venous catheterization during treatment, a history of VTE for ≥1 year, and the absence of anticoagulant therapy were independent risk factors for venous thrombosis in patients with sepsis. The predictive value of the model was further analyzed based on these findings. Our study revealed that the incidence of VTE in patients with sepsis was 4.86%. Currently, data on the incidence of VTE in sepsis patients in China are relatively limited. Internationally, a meta-analysis of adult sepsis across 27 developed countries revealed that the annual incidence of sepsis over the last decade was 4.37% (437/100,000) (20), which is consistent with the results of our study. However, the incidence of VTE among sepsis patients in Chinese ICUs has been reported to be 10%–30% (21), and in our retrospective analysis of 3,228 cases, the incidence of VTE among ICU sepsis patients was 24.5%, which is comparable to the incidence reported by other centers in China. The results of the univariate analysis in this study revealed that bilirubin and creatinine levels, GCS scores, and SOFA scores differed significantly between the VTE group and the non-VTE group. The higher incidence of VTE among ICU patients with sepsis may be due to prolonged bed rest, circulatory failure, mechanical ventilation, intubation, central venous catheterization, and other invasive procedures (22).

The APTT and PT are commonly used in clinical practice to assess coagulation function and represent the functional status of the intrinsic and extrinsic coagulation pathways, respectively. Abnormalities in coagulation function are associated with thrombus formation. The results of our study revealed that APTT, PT, and D-dimer are univariate factors but not independent risk factors for VTE among patients with sepsis. During the modeling process, when these three indicators were included in the prediction model, the calibration curve, CIC, and DCA showed poor fit, indicating that the model had limited predictive power. This may be because coagulation indicators are influenced by surgery, inflammation, and disease severity, resulting in heterogeneity and thus necessitating assessment alongside other indicators.

Our study revealed that the hemoglobin levels in the VTE group (96 g/L) were significantly lower than those in the non-VTE group (109 g/L). However, there were no statistically significant differences in white blood cell or platelet counts between the two groups. A retrospective study on risk factors for VTE in elderly ICU patients found that the Hb levels in the VTE group were lower than those in the non-VTE group, which is consistent with our study results (23). A study on the relationship between hemoglobin levels and VTE in an East Asian population (24) included 6,306 patients with VTE and 2,321 healthy controls and conducted a cohort analysis of their blood cell characteristics. Using Mendelian randomization, the causal relationships between platelet count, hemoglobin level, white blood cell count, and VTE risk were assessed. The results revealed that hemoglobin levels were positively correlated with VTE risk, and platelet count was also positively associated with VTE risk. However, white blood cell count did not demonstrate a significant causal relationship with VTE. A study on hemoglobin levels in patients with sepsis revealed that in elderly patients with sepsis, within 24 h of ICU admission, hemoglobin levels were associated with 28-day mortality risk according to a “U”-shaped curve. Both high hemoglobin levels (≥150 g/L) and low hemoglobin levels (<100 g/L) are associated with increased mortality risk (25). Currently, the causal relationship between hemoglobin levels and the risk of VTE in sepsis is not well established. Our study suggests that low hemoglobin levels may exacerbate ischemia and hypoxia, potentially leading to circulatory disturbances, heightened inflammatory responses, and increased hypercoagulability, which may further promote the occurrence of VTE.

Previously, elevated BMI and increased triglyceride levels were considered high-risk factors for VTE. However, some studies have shown a protective effect of hyperlipidemia against sepsis, which may be attributed to statin use in patients with hyperlipidemia (15). The use of statins is also associated with a reduced risk of sepsis (26). In our study, there was no statistically significant difference in triglyceride levels between the VTE and non-VTE groups.

The results of our study revealed that anticoagulant use was significantly lower in the VTE group than in the non-VTE group. Moreover, the absence of anticoagulant therapy was identified as an independent risk factor for VTE in patients with sepsis. Importantly, the association between the absence of anticoagulant therapy and VTE risk should be interpreted with caution. The decision to withhold anticoagulation may reflect underlying bleeding risk, contraindications, or clinician judgment rather than a pure causal effect. Therefore, our finding should not be interpreted as evidence that anticoagulation should be universally administered to all sepsis patients; rather, it underscores the importance of individualized risk–benefit assessment that balances thrombotic and bleeding risks. The early prevention and treatment of VTE are crucial in clinical practice, similar to the prevention of sepsis, and require a multidisciplinary approach. Treatment modalities include anticoagulation therapy with low-molecular-weight heparin and non-vitamin K antagonist oral anticoagulants (NOACs), the use of elastic compression stockings, and infection control.

A recent systematic review and meta-analysis identified sepsis as an ICU-specific factor with high certainty of association with increased VTE risk (adjusted odds ratio, 1.41; 95% CI, 1.12–1.78) (19). The same meta-analysis also identified central venous catheterization, lack of pharmacologic VTE prophylaxis, and history of recent surgery as significant prognostic factors. A multicenter retrospective study using the MIMIC-IV database similarly identified central venous catheterization as one of the most influential predictors of VTE in septic ICU patients (27, 28). These findings are consistent with our results and provide external validation for the risk factors identified in our model.

Our comparison with established VTE risk assessment tools (Padua Prediction Score and Caprini Risk Assessment Model) demonstrated that our sepsis-specific nomogram provides superior discrimination (AUC 0.909 vs. 0.712 for Padua and 0.738 for Caprini) and significant incremental value as measured by NRI and IDI. This finding underscores the importance of developing sepsis-specific prediction models that incorporate variables unique to the septic population, such as infection site, sepsis severity scores, and inflammatory markers, which are not captured by generic VTE risk assessment tools.

Our study developed a risk prediction model for VTE in patients with sepsis based on the above factors and further validated it. In both the training and validation sets, the ROC and calibration curves showed good model fit, which can help clinical healthcare providers identify risk factors for VTE in patients with sepsis earlier and reduce the occurrence of complications.

This predictive model uses easily obtainable factors in clinical practice, and its algorithm is simple, making it highly feasible to implement in hospitals at all levels. This study has several limitations. First, as a single-center retrospective study, it may be subject to selection bias. There may be some asymptomatic/clinically unsuspected VTE unincluded. Although random sampling was used to select non-VTE controls, the exclusion of 2,368 non-VTE patients may have introduced selection bias. Future studies should consider using the entire non-VTE cohort with class-weighting techniques. Second, although the nomogram model was validated using data from different time periods within our institution over 10 years, external validation using data from other hospitals was not performed. The model was internally validated using a randomly split validation cohort. External validation using data from other institutions is required before clinical implementation. Third, the SMOTE oversampling technique applied to address class imbalance in the training set may have affected model calibration, although our sensitivity analysis suggested that calibration remained acceptable. Fourth, the retrospective nature of the study precluded standardized timing of laboratory measurements between the VTE and non-VTE groups. While we restricted predictor variables to measurements obtained before VTE diagnosis in the VTE group and within 24 h of sepsis diagnosis in the non-VTE group, the exact timing of blood collection was not protocol-driven, which may have introduced heterogeneity. Finally, this observational study extracted data from the database of our center. More precisely designed and planned randomized controlled trials are needed to further analyze and evaluate the risk of sepsis-related VTE before its widespread application.

## Conclusions

In this single-center retrospective cohort study, a sepsis-specific nomogram was developed and internally validated to estimate the risk of VTE among patients with sepsis. The model incorporated four independently associated factors: recent surgery within 3 months, central venous catheterization, history of VTE for ≥1 year, and absence of anticoagulant therapy. Compared with established VTE risk assessment tools (Padua and Caprini scores), the proposed nomogram showed improved discrimination in the validation cohort. However, external validation and prospective impact studies are required before its routine clinical implementation.

## Data Availability

The original contributions presented in the study are included in the article/Supplementary Material; further inquiries can be directed to the corresponding author.
