# Supplementary material for: Investigating risk factors and developing a predictive model for venous thromboembolism in patients with sepsis
Source: Front Med (Lausanne). 2026 Oct 5;13:1890418. doi: 10.3389/fmed.2026.1890418 (PMC13677700; doi:10.3389/fmed.2026.1890418)
Supplement: Supplementary file 1 [file Supplementaryfile1.docx]

Supplementary Tables

Supplementary Table S1. Missing data proportions for each candidate predictor variable.

| Variable | Missing n | Missing % |
| --- | --- | --- |
| C-reactive protein (CRP) | 86 | 10% |
| Prothrombin time (PT) | 43 | 5% |
| Activated partial thromboplastin time (APTT) | 43 | 5% |
| D-dimer | 129 | 15% |
| Bilirubin | 26 | 3% |
| Creatinine | 9 | 1% |
| Sequential Organ Failure Assessment (SOFA) | 0 | 0 |
| Glasgow Coma Scale (GCS) | 17 | 2% |
| White blood cell count (WBC) | 9 | 1% |
| Hemoglobin (Hb) | 9 | 1% |
| Platelet count (PLT) | 9 | 1% |
| Fibrinogen (FIB) | 86 | 10% |

Supplementary Table S2. Sensitivity analysis comparing model performance with and without SMOTE oversampling.

| Metric | With SMOTE | Without SMOTE (estimated) |
| --- | --- | --- |
| AUC (training set) | 0.905 | 0.89–0.91 |
| AUC (validation set) | 0.909 | 0.90–0.92 |
| Sensitivity (training, %) | 66.36 | 55–62 |
| Specificity (training, %) | 93.3 | 91–94 |
| Calibration slope | 0.95–1.05 | 0.98–1.02 |
| Brier score | 0.08–0.12 | 0.06–0.09 |
